# Updates on exposure estimation for the Québec cohort of chrysotile miners and millers: implications for risk assessment and threshold hypothesis

**DOI:** 10.3389/fpubh.2025.1654772

**Published:** 2025-10-03

**Authors:** Bruce W. Case, Andrey A. Korchevskiy, Arseniy Korchevskiy

**Affiliations:** 1 Department of Pathology, McGill University, Montreal, QC, Canada; 2 Chemistry and Industrial Hygiene, Inc., Lakewood, CO, United States

**Keywords:** mesothelioma, chrysotile, Québec cohort, midget impinger, PCM

## Abstract

The McGill University cohort of Québec chrysotile miners and millers (total of 11,379 members) has been a major determinant of risk assessment. The U. S. EPA and some other meta-analyses omit this cohort, the largest ever exposed to high concentrations of chrysotile. Preliminary analyses using new data showed average cumulative exposures in the full cohort of 528.5 f/cc-years (95% CI 510.3, 546.7) or 979 f/cc-years (95% CI 945, 1,013), depending on the regression equation used for the modeling. The lowest estimated exposure level observed for any mesothelioma case with diagnostic certainty was 68 f/cc-years. We confirmed that the estimation of potency factor for Québec chrysotile should not be expected to differ significantly from Darnton’s estimation for non-textile chrysotile (R_M_ = 0.0014%), if better quality data for correspondence between impinger and fiber count measurements is involved. Among several estimations we made, the average meta-analytical mesothelioma potency factor for all types of chrysotile would be 0.0014% (95% CI 0.0010, 0.0.0018), and for non-textile chrysotile 0.0011% (95% CI 0.0008, 0.0015). Several estimations of a possible threshold that would correspond to non-elevated mesothelioma risk based on the Québec cohort were also made. The minimal cumulative exposure in mesothelioma cases demonstrated values that ranged from 4.8–6 MPCF-years, or 18–50 f/cc-years. Also, based on theoretical models, the threshold can be found at the level of 6.38 MPCF-years (standard deviation 2.6), or in the range from 35.9 f/cc-years (standard deviation of 15.4) to 65.9 f/cc-years (standard deviation of 29.6).

## 1 Introduction

The Québec miners and millers cohort study (1–4) is one of the most important sets of epidemiological evidence for the study of asbestos health effects, including potential differences between chrysotile and the asbestiform amphiboles’ carcinogenic potential and mesothelioma potency.

At the same time, methodology and quality of exposure estimations for this cohort has been questioned (5). Recently, the U. S. EPA (6) excluded Québec data from its potency estimation for chrysotile, potentially causing significant bias in the meta-analysis because the largest data source was ignored. Darnton (7) demonstrated that exclusion of Québec information from mesothelioma meta-analysis can artificially elevate the potency factor for chrysotile. Burdett (8) argued that exclusion of the Québec cohort does not seem reasonable given the plausible extent to which this data point could be errant, and doing so would likely bias the chrysotile risk estimation upwards considerably”.

In fact, the Québec cohort has consistently been that with the largest absolute numbers of mesothelioma deaths among chrysotile-exposed cohorts. Given the historic nature of the cohort, “comprising all those born between 1891 and 1920 who had ever been employed in any part of the industry for more than 1 month” (1), it is not surprising that the confidence with which deaths were ascribed to mesothelioma varied, as did possibilities for alternative asbestos exposures. As of 1972, seven cases had been identified, but one was rejected by the Canadian Mesothelioma Panel of Pathologists, a second probably had domestic exposure exceeding his cohort exposure, and a third had an uncertain history of having ever worked in the industry at all. All were nonetheless included in cohort study and carried forward as mesothelioma deaths in the cohort. By the end of 1975, 10 male and one female death due to pleural mesothelioma had been identified, nine from death certificates and one at necropsy (2), of a total number of 4,463 male deaths in a cohort of 10,939 and 84 deaths among 440 eligible women. By the end of 1988, there were 33 deaths from mesothelioma in the cohort, 28 in miners and millers and five employees of a small asbestos products factory where commercial amphibole asbestos had also been used (3). As of final publication, there were overall 8,009 deaths in the cohort to end 1992, and five additional mesothelioma deaths had been added (4).

Like the 1973 assessment, the 1997 cohort analysis was inclusive for all *possible* cases of mesothelioma. The total of 38 cases (including five from the Johns-Manville factory where crocidolite was used) included *all* deaths in which the term mesothelioma was mentioned on the death certificate, *and all* deaths coded from 1978 to ICD 163 (cancer of pleura); by ongoing ascertainment of malignant mesothelioma through all Canadian pathologists, 1966–1984; and by including data from more recent studies by other Québec investigators with special interest in mesothelioma. Diagnosis was “confirmed by full autopsy in 27 cases, but in eight of these the pathologist expressed an element of doubt.” Overall, the “admittedly subjective assessment of the diagnosis being correct was high in 19, moderate in 14, and low (though more likely than not) in five”.

As noted by Goodman et al. (9), diagnostic error for mesothelioma can contribute to misclassification of risk in either direction. Although, as noted, pathology reports—most often autopsy-based—were available for the Québec cases, they relied histologically on Hematoxylin and Eosin (H & E) staining, since no cases were diagnosed after 1992. More recent studies have been improved by the advent, in the late 1990s, of immunohistochemical (IHC) markers of mesothelial cell origin, although these vary in sensitivity and specificity. The gradual evolution and improvement in certainty of pathology diagnosis attributable to IHC advances is described elsewhere (10).

In addition to diagnostic doubt, a significant impediment to data quality assessment for the Québec cohort lay in the fact that the majority of the exposure assessment for chrysotile miners and millers had necessarily been performed based on the original midget impinger dust measurements available at the time, and not on actual fiber counts. Berman and Crump (11, 12) assumed that there is a constant ratio that could be applied to the Québec impinger concentrations to derive the fiber concentration in f/cc. This coefficient was assumed to be equal to 3. However, as Burdett (8) noted, the utilization of a ratio value for midget impinger and Phase Contrast Microscopy (PCM) fibers would require additional substantiation. Also, Robock, (13), expressed doubts about the methodological feasibility of recalculating midget impinger concentration to PCM (f/ml, or f/cc) estimations. Robock correctly asserted that the range of the ratio between PCM and midget impinger concentrations varied significantly by industry, or even at the same location. Certainly, a relationship between fiber concentration by PCM and midget impinger measurements exists objectively: one should increase with another. However, there are numerous confounding factors for this relationship. It is expected that additional studies are beneficial to see if the objective relationship between the two methods can be approximated using available data, at least for a certain location (as in the Québec mining and milling cohort study).

The purpose of this paper is to explore whether use of an expanded set of now available original data for parallel measurements of asbestos concentrations in the Québec mines and mills in MPCF and in fibers/cc supports average estimation of cumulative exposure as used in the meta-analysis recently updated by Darnton (7, 14). While there are difficulties in reconstructing cumulative exposure in PCM units for every person in the cohort (because of limited numbers of parallel measurements and the presence of confounders), we can demonstrate that estimations of average exposure for all cohort members can be made with good predictive quality.

We demonstrate that application of strict quality-based criteria to mesothelioma cases in the study also helps to confirm estimations of chrysotile potency as published by Darnton. We use the original set of chrysotile datapoints from Darnton (7, 14), supplementing it with recently published updated exposure values for Balangero miners and millers (15), as well as from the new IARC publication on mesothelioma in chrysotile asbestos miners and millers in Russia (16). We test the changes observed in the meta-analysis performed by Darnton if newly-derived estimations of average cumulative exposure in Québec miners and millers are used.

We also explore estimations of minimal cumulative exposure in confirmed mesothelioma cases and show that there is no data on any individual mesothelioma in the Québec cohort with chrysotile exposure lower than a certain value. Fully proving the existence of a threshold for mesothelioma is an impossibility so long as a linear no-threshold model is adhered to; in the words of Hodgson and Darnton (17), this is a “logical nonsense.” However, a lowest observed adverse effect level for diagnostically confirmed mesothelioma cases. Without known alternative asbestos exposures and with the enhanced exposure assessments now available among the 8,009 deaths of chrysotile workers in this cohort may serve as a “practical” guide, informing toxicological studies and regulatory efforts.

We also attempt the use of several different models that also support the concept of the existence of a chrysotile exposure range not associated with excess mesothelioma risk.

It should be emphasized that this paper is focused on a very important topic of historical exposure data reliability. Human epidemiology provides the most critical part of evidence for carcinogenicity of various agents. Burdett (8) reviewed the reliability of risk estimations based on historical data for workers’ exposure to various mineral types of asbestos. Burdett demonstrated that consistency of the results of meta-analysis for crocidolite, amosite, and chrysotile, as demonstrated in major studies during the recent decades, can be considered the evidence of reliability of exposure information. In this paper, we will explore the role of method-to-method conversion (in this case, midget impinger to optical microscopy) for deriving mesothelioma slope factors. We will use several new approaches (including statistical simulation of possible uncertainty of average exposure estimates vs. individual datapoints) to substantiate the reliability of historical data assessment. We will also demonstrate how minimal reported exposure associated with the cases of disease can be estimated and how it can be used for derivation of “practical threshold” for chrysotile asbestos.

## 2 Materials and methods

### 2.1 The cohort

#### 2.1.1 Introduction

The study of the Québec asbestos mining and milling cohort came about after Dr. J. Corbett McDonald, the newly installed Chair of Epidemiology at McGill University, attended the 1964 New York Conference (18) at the suggestion of Dr. J. C. Wagner (19). Following the conference, 40 delegates from eight countries formed a Working Group (October 22–23, 1964). A Report and Recommendations (20, 21) were prepared. Under “Terms of Reference,” the first recommendation [(21), pg. 165] was:

*“I. Epidemiology: A. To investigate the incidence of mesothelial tumours of the pleura and peritoneum in groups and/or regions where exposure to only one type of asbestos fibre has occurred.”*

The UICC Working Group (20) specifically suggested that chrysotile studies be undertaken in Canada, and in March 1965, McDonald joined a National Study Group on Asbestos convened by the Canadian Department of National Health and Welfare. He was asked to serve as Principal Investigator for epidemiological studies; shortly thereafter the study group was formed and the cohort defined.

#### 2.1.2 Subjects

McDonald et al. (4), with data on mesothelioma diagnosis certainties, along with indication of possible alternative amphibole asbestos exposures (crocidolite, amosite, asbestiform tremolite), identified 38 mesothelioma deaths among 8,009 cohort deaths. Five cohort members with history of factory work were excluded from consideration due to reported amphibole asbestos exposure, along with six mesothelioma cases with short term work (2–5 years) in the mines and mills and longer lifetime other employment or domestic asbestos exposure(s).

For mesothelioma mortality assessment, an archived, unpublished dataset prepared at Imperial College, London by Professor Douglas Liddell, based on the original cohort data, and provided to Berman and Crump for their studies of mesothelioma risk was used. The dataset, which lacked individual identifying data, included 9,244 cohort members and their exposure intensity (in MPCF, by midget impinger), duration, age at death, interval between end of employment and death, and mesothelioma as cause of death (or not) included. The dataset was compared with the published information for mesothelioma cases (4), which included mesothelioma diagnosis certainty and indication of possible alternative occupational or domestic asbestos exposure among short-term workers (2–5 years). A total of 35 mesothelioma deaths were present in both Liddell’s dataset as provided to Berman and Crump and the published information on the 8,009 deaths in the cohort (4).

All five cases with a history of factory work (4, 22) were also excluded from consideration due to work history time period and/or lung fiber content indicating crocidolite exposure in the manufacture of gas masks. This left a qualifying sub-cohort of 8,486 persons, with 30 cases of mesothelioma.

For three cases among the 30, diagnostic probability was low in the original study (4); these were excluded when indicated in the study.

Overall, this left 27 mesothelioma cases as at least “moderately” confirmed pathologically, though exclusion of an additional four cases may be warranted as well.

We consider this approach to excluding cases as conservative, but we believe it provides us with the best possible estimation of true mesothelioma cases to relate to chrysotile exposures.

### 2.2 Exposure and mesothelioma risk assessment

We obtained the document published by M. Dagbert (23) containing the original data for parallel measurements of midget impinger and PCM fiber counting methods. A total of 621 paired datapoints were listed by Dagbert; each pair had codes for mine, department, site, and sample #.

We used a log–log regression equation to characterize the relationship between fiber concentration and midget impinger concentration:


$$\log_{10}(F)=A\times \log_{10}(\mathrm{MI})+B,$$ (1)

where F—concentration of fibers longer than 5 μm by PCM (f/cc), and MI—midget impinger concentration of fibers (MPCF).

Assuming that close levels of midget impinger exposure are expected to result in close levels of fiber concentrations, we grouped consequent values of concentrations in the Dagbert data and averaged each result. It allowed us to get better correlation between MI and F variables. Though in general combining datapoints may not be a fully justified way to determine the regression between variables, we assumed that this approach is reasonable for comparison of two exposure assessment methods where the relationships between variables is assumed to objectively exist, while being potentially obscure by precision of each method. Combining consequent datapoints in this case would allow for improved prediction power, rather than just reducing the variability of each of the parameters. The grouping was done for each of the 30 consequent values of impinger measurements, comprising approximately 5% of the data in each group.

We calculated cumulative exposure for workers in our study by the equation:


CE=CxD,

where CE—cumulative exposure, measured in f/cc-years or MPCF-years, C—average exposure concentration (f/cc or MPCF), D—exposure duration (years).

Cumulative exposure in f/cc-years is widely considered as a metric of lifetime carcinogenicity in asbestos exposure (7, 14, 17).

Concentration C for subjects in the study were estimated in MPCF in a dataset described above, according to McDonald et al. (4) based on available individual exposure history. It is assumed that C is a good representation of exposure time weighted average (TWA) for daily exposure, as well as for lifetime exposure estimations. The methods to recalculate CE from MPCF-years to f/cc-years are discussed in this paper.

Mesothelioma potency factor *R*_M_ for this study was estimated by the Hodgson and Darnton method using the following formula:


$$R_{M}=100\frac{O_{M}}{\left(E_{\mathrm{ADj}}\times \mathrm{CE}\right)},$$

where *O*_M_ is the number of mesothelioma deaths, *E*_Adj_ the total expected deaths from all causes adjusted to the age of first exposure of 30, and CE is the mean cumulative exposure in phase-contrast microscopy (PCM) fibers with length > 5 μm/cubic centimeter-years (f/cc-years). The definition of PCM fibers in the Hodgson and Darnton (17) method uses length criteria for countable fibers (length > 5 μm). PCM fibers also have aspect ratio > = 3:1 and minimal width of fibers, depending on microscopy resolution and mineral type of fibers (currently assumed to be 0.15 μm for chrysotile and 0.05 μm for amphiboles) (NIOSH PCM Issue 3) (24).

It should be also noted that the Hodgson and Darnton method utilized single point estimations of the mean cumulative exposure for each cohort in their meta-analysis, to provide for overall estimation of the average potency factor, considering potential uncertainty of exposure estimations and difficulties in comparing data from various sources (7, 14). We were interested in checking how Dagbert’s original data would affect the average estimation of X for the Québec cohort, and if it would change significantly the level of R_M_ for this cohort.

For our study, we assumed that total expected death from all causes in Québec workers is a function of average age of the sub-cohort and cohort size. Based on Darnton (7), we assumed that total expected mortality is about 59.6% of the cohort size for Québec workers, if the average age of the cohort at the observation end is about 63.5 years. Darnton assumed that the average exposure onset age of Québec workers was close to 30 years, and no correction for the age of first exposure was needed (coefficient of 1.0). We assumed the same for the sub-cohorts in our study.

Several changes were made to further improve the estimations of potency provided in Darnton, 2023 (14). In particular, a new estimation of the average cumulative exposure for the Balangero cohort was made based on the recent publication on the matter (15). Also, a Russian cohort was added to the dataset based on a recent IARC publication.

The data for meta-analytical potency estimation is provided in Table 1.

**Table 1:** Data for chrysotile potency estimation.

Cohort	Textile (T) or non-textile (NT)	Mesothelioma cases	Total expected mortality	Age adjustment factor	Cumulative exposure (f/cc-years)	Average R_M_, %
Québec	NT	33	5912.7	1	600	0.0009
Balangero	NT	7	533.4	1.03	700	0.0018
Qinghai	NT	0	174.2	2.1	120	0
New Orleans (plant 2)	NT	0	397.1	1.26	22	0
South Carolina (women)	T	0	549.6	1.21	26	0
South Carolina (men)	T	3	571.1	1.21	28	0.016
North Carolina	T	8	1275.3	0.94	68.3	0.0098
Chongqing factory	T	2	197.3	0.43	105.2	0.022
Connecticut	NT	2	2,800	0.98	46	0.0016
Russia	NT	13	11,503	1	33.6	0.0033
Total chrysotile						0.0014
Total non-textile						0.0011

We estimated cumulative exposure to fibers for each cohort member in our study by applying Equation 1 (with fitted coefficients) to the reported individual exposure intensity in MPCF and multiplying the result by exposure duration in years.

We performed a statistical simulation to check if regression equations would adequately predict average values for fiber exposure. For this purpose, we selected random combinations of datapoints from the Dagbert dataset. Combinations of 30 to 100% of datapoints were used. The actual average values were found for each combination of datapoints and compared to the average of the values predicted by Equation 1. Also, from each combination, average was found for 20% of the lowest levels of impinger concentrations, as well as 20% of the highest levels. This allowed us to see how the relationship between observed and predicted levels of exposure would change with increasing concentrations.

We used statistical modeling to estimate changes in estimation of potency for all chrysotile cohorts and non-textile cohorts that would be caused by changing the improved estimation of cumulative exposure, number of mesothelioma cases and total expected mortality for the Québec cohort. To calculate meta-analytical average potency levels, Poisson regression was used as in the Hodgson and Darnton method [(17); and Darnton personal communication in 2025].

To model mesothelioma rate as a function of cumulative exposure, we performed a statistical simulation. For each iteration, four random intervals of cumulative exposure (measured in MPCF-years or f/cc-years) were selected, with not less than 500 datapoints for each interval. The following regression equation was found between mesothelioma rate and log-transformed cumulative exposure at each interval:


$$\text{Meso}=A\times \log_{10}(\mathrm{CE})+B,$$ (2)

where Meso—mesothelioma rate per person for a selected cumulative exposure interval, and CE—cumulative exposure, measured in MPCF-years or f/cc-years based on the conversion method.

A total of 10,000 iterations were performed. The regression with the highest correlation coefficient was selected. Because of the linear-log regressions were applied, non-zero threshold existed for each of the regressions:


$$\text{Threshold}=10^{-B/A}.$$ (3)

The methodological rational in using Equation 3 for determination of threshold is based on the shape of relationship in Equation 2. Cancer rate as a function of log-transformed exposure is a widely used curve for dose–response in carcinogenicity (25).

At a cumulative exposure lower than threshold, negative mesothelioma rate could be predicted, indicating a transition between different stages when mesotheliomagenic effect would be absent or present.

This approach would not be able to prove the existence of threshold but can approximate its level in a case when regression in Equation 2 would demonstrate the highest fit to the data.

Also, we used the data from McDonald et al. (4) to evaluate chrysotile and tremolite lung concentrations in mesothelioma cases, included in our analysis, that we juxtaposed with cumulative exposure values. For chrysotile and tremolite, lung concentration was available for 15 subjects as obtained using the methodology specified in Case et al. (26).

Statistica 14.0 software was used for statistical calculations and visualization. A software developed in Python was used to perform statistical simulations.

## 3 Results

### 3.1 Relationship between PCM fiber concentration and midget impinger measurements

The Liddell data provides a wide variety of datapoints for various levels of duration (in years) and intensity (in MPCF).

**Figure 1:**
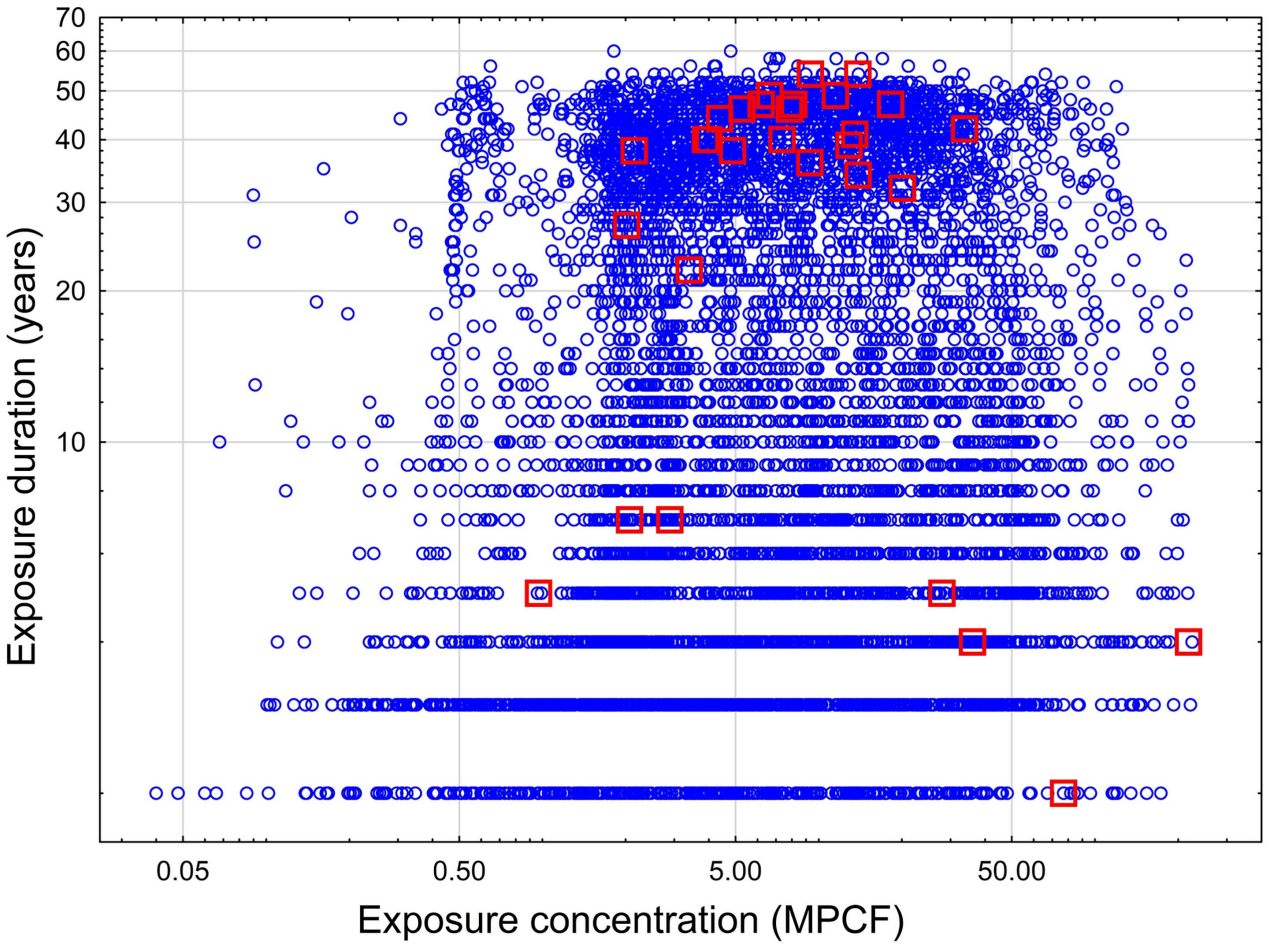
Concentration and duration of exposure for workers in a cohort study. Blue circle – no mesothelioma, red squares – mesothelioma cases.

The two-dimensional distribution of concentration and duration of exposure is illustrated in Figure 1.

As we can see, most of the mesothelioma cases are concentrated in the area with duration higher than 20 years and exposure intensity higher than 2 MPCF. The second group of mesothelioma cases is associated with exposure intensity higher than 27.9 MPCF, but with lower duration (probably, at this level of exposure the effect depended on exposure intensity, but not duration). Also, three cases of mesothelioma comprised a third group with lower intensity (from 0.9 to 2.8 MPCF) and duration (from 5 to 7 years). This group may represent some outlying combination of exposure level and confounding factors.

The Dagbert data (23) provides information about PCM fiber concentrations measured in parallel with impinger measurements. The data is available for impinger measurements in the range from 0.04 to 9.12 MPCF.

Based on this data (23), the following relationship was discovered for all mines, departments, and sites combined:


$$\log_{10}(F)=0.71\times \log_{10}(\mathrm{MI})+0.76,$$ (4)

where F—concentration of fibers longer than 5 μm by PCM (f/cc), and MI—midget impinger concentration of fibers (MPCF) (*R* = 0.46, *R*^2^ = 0.21, *p* < 0.00001).

While the correlation in Equation 4 is weak, it is still statistically significant.

We also determined the following regression equation by averaging F and MI values for every 30 datapoints of a sorted MI variable (sorting from lowest to highest values):


$$\log_{10}(F^{*})=0.73\times \log_{10}({\mathrm{MI}}^{*})+1.01,$$ (5)

where F*—concentration of fibers longer than 5 μm by PCM, averaged for 30 datapoints (f/cc), and MI*—midget impinger concentration of fibers, averaged for each 30 datapoints (MPCF) (*R* = 0.91, *R*^2^ = 0.82, *p* < 0.000001).

The grouping was done for each of the 30 consequent values of impinger measurements, comprising approximately 5% of the data in each group. The averaging allowed to filter potential “noise” in the data, helping to elucidate a relationship between variables existing.

Both Equations 4, 5 can provide information about the true relationship between F and MI, assuming that this relationship exists objectively. Equation 5 would systematically provide higher estimations of F for a given MI.

**Figure 2:**
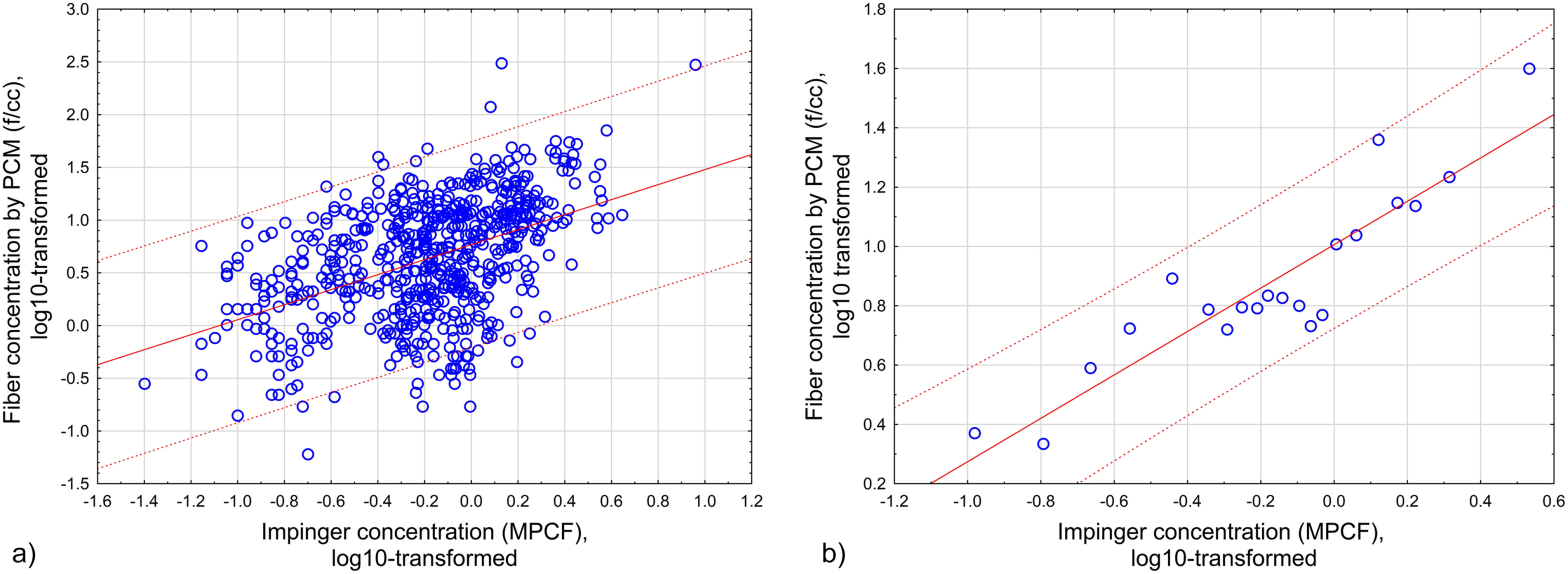
Approximation of log_10_-transformed fiber concentration (f/cc) by log_10_-transformed midget impinger concentrations: **(a)** All original datapoints. **(b)** Grouped datapoints. Red lines -regression relationships. Dotted lines – 95% CI. **(a)** All original datapoints (Equation 4; *R* = 0.46, *R*^2^ = 0.21, *p* < 0.00001). **(b)** Grouped datapoints (Equation 5; *R* = 0.91, *R*^2^ = 0.82, *p* < 0.000001).

Figure 2 illustrates approximation of log_10_-transformed fiber concentration (f/cc) by log_10_-transformed midget impinger concentrations - Figure 2a for Equation 4, Figure 2b for Equation 5.

### 3.2 Estimation of the validity of the averaged exposure levels estimated by regression equations

As was indicated in the Materials and methods section, a statistical simulation was performed to check if regression equations would adequately predict average values for fiber exposure. For this purpose, we selected random combinations of datapoints from the Dagbert dataset. Combinations of 30 to 100% of datapoints were used. We utilized Monte Carlo simulation with 10,000 attempts. The actual average values were found for each combination of datapoints and compared to the average of the values predicted by Equation 1. Also, from each combination, average was found for 20% of the lowest levels of impinger concentrations, as well as 20% of the highest levels. This allowed us to see how the relationship between observed and predicted levels of exposure would change with increasing concentrations. The validity of the simulation is supported by the high number of iterations.

**Figure 3:**
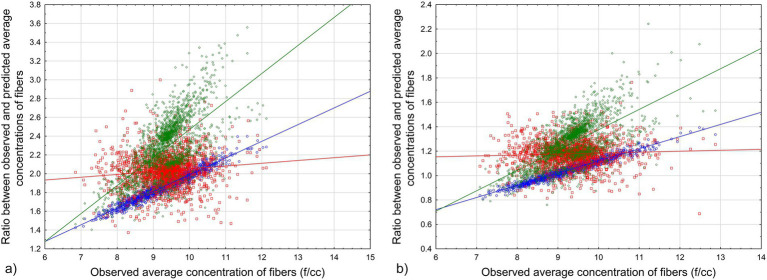
Ratio between observed and predicted average values for various combinations of Dagbert datapoints: **(a)** Equation 4. **(b)** Equation 5. Blue circles – full range of data, red squares – lowest 20%, green rhombuses – highest 20%. Lines – corresponding regression lines of the relationship between average exposure in MPCF and the ratio.

The results of the simulation for estimated average values of observed fiber concentrations vs. values predicted by Equations 4, 5 are demonstrated in Figure 3.

The results of the simulation show that for every combination of datapoints from Dagbert dataset (reflecting possible measurements for workers in the database), the modeled average cumulative exposure of workers will be a good representation of the true average in f/cc-years that would be observed in the cohort.

We can see from the results of the simulation that average cumulative exposure by Equation 4 will underestimate the observed exposure level; it means that potency factors would be overestimated with Equation 4. Equation 5 produced closer estimations of observed exposure, with the ratio between observed and predicted exposure being not less than 0.8 (except maybe for outliers).

### 3.3 Estimation of chrysotile potency factor in Québec cohort with various assumptions

Table 2 contains data and estimations of R_M_ potency based on various calculation scenarios. We referred to the original study as used by Berman and Crump (11, 12), Hodgson and Darnton (17), and Darnton (7, 14). We also utilized scenarios with Equations 4, 5 used for midget impinger to PCM fiber concentrations recalculated, applying the models to all mesothelioma cases, or only to cases with acceptable diagnostic quality. Besides that, for every approach used, we determined the minimal level of exposure associated with a mesothelioma case. Also, meta-analytical mesothelioma potency factor R_M_ (%) was calculated for total chrysotile exposure and total non-textile cohorts.

**Table 2:** Results of the cumulative exposure estimations in Québec workers for various scenarios.

No.	Scenario	Total expected mortality	Average cumulative exposure (f/cc - years)	Number of mesothelioma cases	Minimal exposure associated with mesothelioma (f/cc-years, and/or MPCF)	Estimation of R_M_ (%)	
						All cohorts	All non-textile cohorts
1.	Original Liddell, Berman, Crump	5912.7	600	33	6 MPCF, 18 f/cc-years	0.0014	0.0011
2.	Exposure estimation based on Equation 4						
	(a) All mesothelioma cases	5,057	528.5	30	4.8 MPCF, 28.6 f/cc-years	0.0017	0.0014
	(b) Cases with low diagnostic confidence excluded	5,057	528.5	27	4.8 MPCF, 28.6 f/cc-years	0.0017	0.0014
3.	Exposure estimation based on Equation 5						
	All mesothelioma cases	5,057	979.3	30	4.8 MPCF, 50 f/cc-years	0.0011	0.0009
	Cases with low diagnostic confidence excluded	5,057	979.3	27	4.8 MPCF, 50 f/cc-years	0.0010	0.0008

For Equation 4, the average cumulative exposure for the cohort was estimated at 528.5 f/cc-years (95% CI 510.3, 546.7), and for Equation 5, it was found equal to 979 f/cc-years (95% CI 945, 1,013).

As can be seen, the application of Equation 4 to this data demonstrates that with additional inclusion of Russian cohort to the meta-analysis, the potency factor for all types of chrysotile slightly exceeds Darnton’s estimation (0.0017 vs. 0.0014%). The average R_M_ for non-textile cohort in Darnton (14) was estimated as 0.0011%, consistent with the range of estimations in our analysis based on Equation 4.

If Equation 5 were utilized, R_M_ for chrysotile would be lower than in Darnton’s analysis: 0.0010% in our estimation vs. 0.0014% in Darnton. For non-textile cohorts, our approach brings an even lower value: 0.0008%.

Among several estimations we made, the average meta-analytical mesothelioma potency factor for all types of chrysotile would be 0.0014% (95% CI 0.0010, 0.0.0018), and for non-textile chrysotile 0.0011% (95% CI 0.0008, 0.0015).

It should be noted that, as we demonstrated, regression equations predict reasonable lower bound for average exposure (see 3.2).

We can treat our estimation of minimal exposure associated with mesothelioma as the “practical threshold,” or Lowest Observed Adverse Exposure Level (LOAEL). Estimations of minimal exposure in PCM units range from 18 f/cc-years, if all 30 mesothelioma deaths are included (as in the original study by Liddell), up to 28–50 f/cc-years, if we exclude cases with possible low diagnosis confidence and rely on Dagbert’s data for exposure calculations.

### 3.4 Modeling of mesothelioma risk as a function of cumulative exposure

The data for mesothelioma mortality in the Québec cohort can be empirically subdivided by various subgroups to demonstrate the dose–response relationship and the shape of dose–response curve.

We performed a simulation to find cumulative exposure and mesothelioma risk at different intervals, starting with minimum, and ending with maximum value of exposure. For each iteration, four random intervals were selected, with not less than 500 datapoints for each interval. The Monte Carlo method was used for the selection of combinations, with 10,000 iterations performed. Regression equation was found between mesothelioma rate and log-transformed cumulative exposure at each interval. For each modeling attempt, all 30 mesothelioma cases were included in the analysis.

The following intervals produced the highest correlation of mesothelioma rate with cumulative exposure.

Based on midget impinger measurements, the intervals were determined to generate regression relationship with *R* = 0.999, *R*^2^ = 0.999, *p* < 0.000066. The data is shown in Table 3.

**Table 3:** Relationship between mesothelioma mortality and cumulative exposure by midget impinger.

Mesothelioma rate per person	Average cumulative exposure (MPCF-years)	Average age (years)	Number of persons
0.0009	14.9	59.3	4,433
0.0037	71.6	62.7	546
0.0047	124.9	63.6	1,070
0.0078	690.8	66.2	2,437

The following regression (Equation 6) was developed:


MesoRate=0.0041log10(CE)−0.0039, (6)

where MesoRate—lifetime mesothelioma rate per person, and CE—cumulative exposure (MPCF-years).

**Figure 4:**
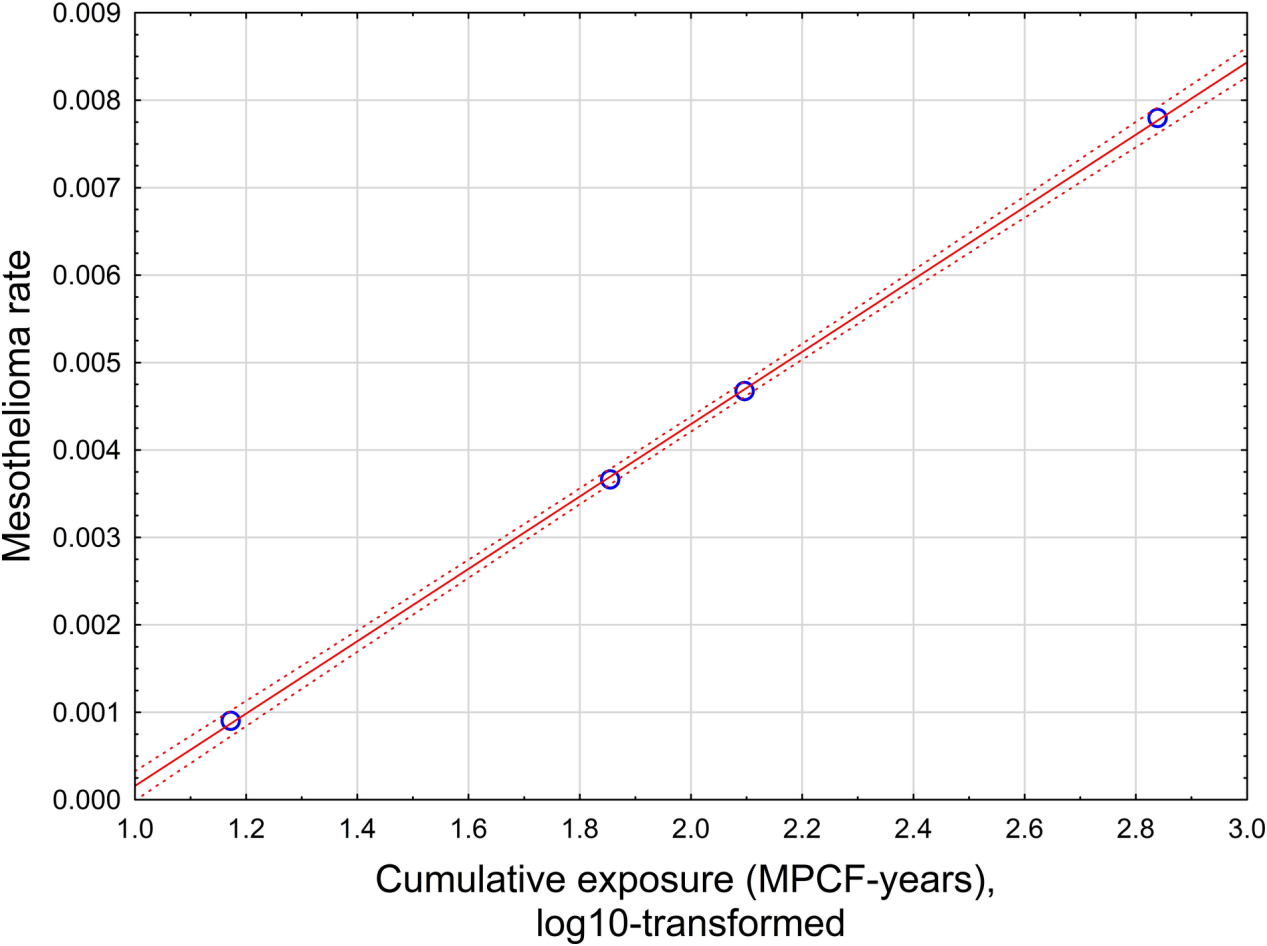
The relationship between mesothelioma rate and cumulative exposure, estimated by midget impinger.

The relationship is illustrated in Figure 4.

With high statistical confidence, this relationship is supporting the level of positive threshold of 8.9 MPCF-years.

If all possible combinations of intervals would be considered, assuming *p* < 0.05 for dose–response regression, the average threshold value can be found at the level of 6.38 MPCF-years with standard deviation of 2.6 (total of 780 regression with statistically significant dose–response, from 10,000 total regressions tested).

Based on Table 3, age at the end of observations is also a strong predictor of mesothelioma rate.

For example,


$$\text{MesoRate}=0.14\;\log_{10}(\mathrm{Age})-0.25,$$ (7)

where MesoRate—lifetime mesothelioma rate per person, and Age—the age at the end of observations (*R* = 0.99, *R*^2^ = 0.98, *p* < 0.009).

However, it is most likely that age in Equation 7 reflects the level of cumulative exposure, rather than playing a separate role in mesothelioma rate prediction. If age and cumulative exposure (by midget impinger) were both included in regression equation, age would have a negative coefficient (that is unreasonable).

Based on Equation 4, the intervals were found to generate regression relationship with *R* = 0.999, *R*^2^ = 0.999, *p* < 0.00036. The data is shown in Table 4.

**Table 4:** Relationship between mesothelioma mortality and cumulative exposure by Equation 4.

Mesothelioma rate per person	Average cumulative exposure (f/cc-years)	Average age (years)	Number of persons
0.0010	24.2	59.1	5,067
0.0048	126.2	64.4	829
0.0066	265.4	66.6	1,209
0.0094	994.9	67.1	1,381

The following regression (Equation 8) was developed:


$$\text{MesoRate}=0.0052\;\log_{10}(\mathrm{CE})-0.0062,$$ (8)

where MesoRate—lifetime mesothelioma rate per person, and CE—cumulative exposure, based on Equation 4.

**Figure 5:**
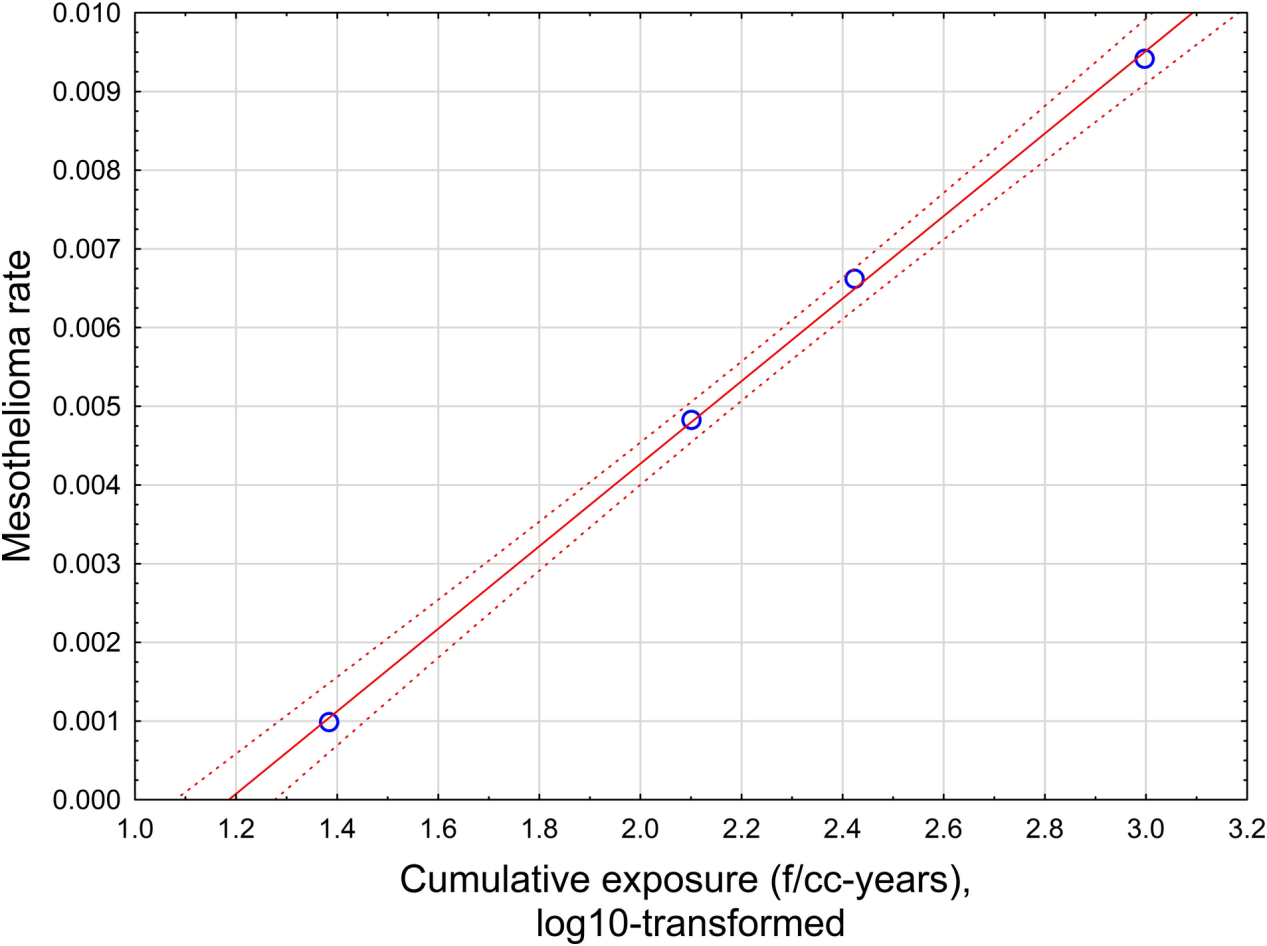
The relationship between mesothelioma rate and cumulative exposure is estimated by Equation 4. Red solid line – regression equation, dotted lines −95% confidence interval.

The relationship is illustrated in Figure 5.

This relationship supports a level of positive threshold of 15.8 f/cc-years with a range from 12.6 to 19.95 f/cc-years.

If all possible combinations of intervals are considered, assuming *p* < 0.05 for dose–response regression, the average threshold value can be found at the level of 35.9 f/cc-years with standard deviation of 15.4 (total of 781 regressions with statistically significant dose–response, from 10,000 total regressions tested).

Based on Equation 5, the intervals were found to generate regression relationship with *R* = 0.999, *R*^2^ = 0.999, *p* < 0.00001. The data is shown in Table 5.

**Table 5:** Relationship between mesothelioma mortality and cumulative exposure by Equation 5.

Mesothelioma rate per person	Average cumulative exposure (f/cc-years)	Average age (years)	Number of persons
0.0007	90.2	58.6	4,218
0.0043	499.8	62.7	1,393
0.0062	1278.5	66.5	1,601
0.0086	4071.3	66.9	1,274

The following regression equation was developed


$$\text{MesoRate}=0.0047\;\log_{10}(\mathrm{CE})-0.0086,$$ (9)

where MesoRate—lifetime mesothelioma rate per person, and CE—cumulative exposure, based on Equation 5.

**Figure 6:**
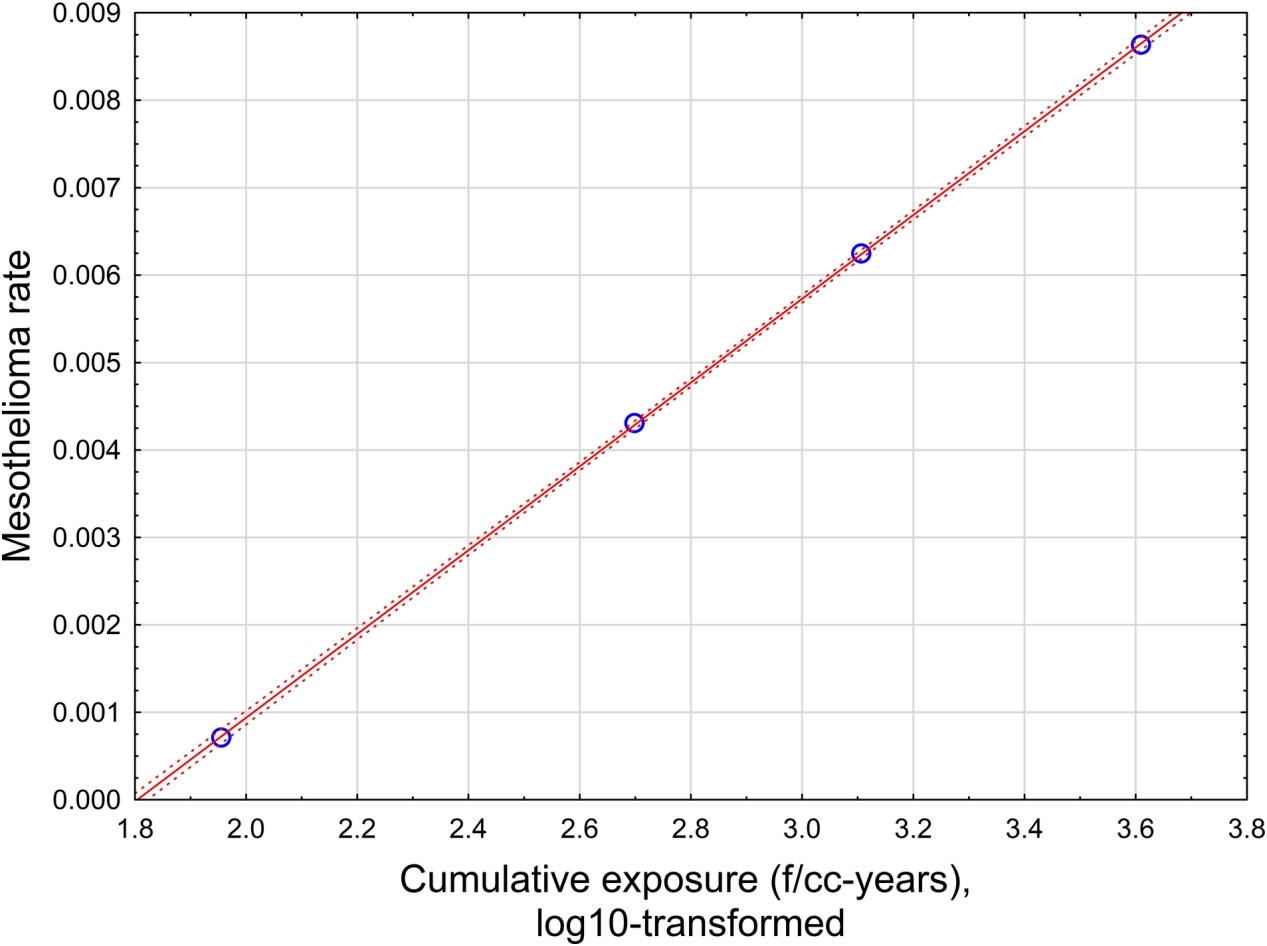
The relationship between mesothelioma rate and cumulative exposure is estimated by Equation 5. Red solid line – regression equation, dotted lines −95% confidence interval.

The relationship is illustrated in Figure 6.

With high statistical confidence, this relationship is supporting the level of positive threshold of 67.6 f/cc-years.

If all possible combinations of intervals are considered, assuming *p* < 0.05 for dose–response regression, the average threshold value can be found at the level of 65.9 f/cc-years with standard deviation of 29.6 (total of 862 regressions with statistically significant dose–response, from 10,000 total regressions tested).

### 3.5 Testing mesothelioma risk for random combinations of datapoints

We also tested the relationship between mesothelioma rate and cumulative exposure in Québec cohort by selecting random combinations of persons in the Liddell database and calculating average cumulative exposure values by impinger measurements and by Equations 4, 5. Mesothelioma rate was calculated as probability of a mesothelioma case in the selection. Size of subgroups varies from 0.1 to 100% of the whole cohort.

**Figure 7:**
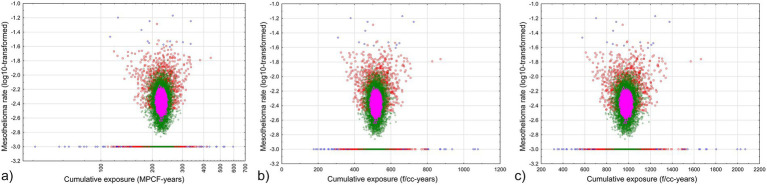
Relationship between average cumulative exposure and mesothelioma rate in random selections from the Québec dataset. Blue circles – fraction of persons less than 0.5% of total cohort, red squares – fraction of persons less than 5% of total cohort, green rhombuses – fraction of persons less than 50% of total cohort, magenta triangles - fraction of persons more than 50% of total cohort. **(a)** midget impinger **(b)** PCM by Equation 4 **(c)** PCM by Equation 5.

The results of the simulation are illustrated in Figure 7. Mesothelioma rate was log-transformed, with adding 0.001 to each value before logarithm was taken, to demonstrate zero values along with selections with some mesothelioma cases.

As we can see, with virtually all range of cumulative exposure to chrysotile asbestos, zero mesothelioma rates can be a possibility. No mesothelioma cases are determined for average cumulative exposure levels of 113 MPCF-years by impinger, 309 f/cc-years by PCM Equation 4, or 577 f/cc-years by Equation 5. It means that even with lower individual threshold levels of estimated cumulative exposure, more reliable values of cumulative exposure, averaged by groups, shows that considerably high level of exposure is required to elevate corresponding average mesothelioma rates.

We also can see that only one group of subsamples always has non-zero mesothelioma risk: it is the selections with fraction more than 50%, and with cumulative exposure from 206 to 240 MPCF-years, or 480 to 559 f/cc-years by Equation 4, or 927 to 1,058 f/cc-years by Equation 5. There are no magenta triangles for these ranges at the lines at Figure 7 indicating zero mesothelioma risk. It means at chrysotile exposure levels below 480–927 f/cc-years, mesothelioma can randomly occur; only starting with the level of 480–927 f/cc-years mesothelioma is definitely caused by exposure; the risk obviously will be not lower for higher exposure, but the reliability of the estimations drops (because only smaller selection size is associated with very high exposure).

### 3.6 Relationship between fiber lung burden and cumulative exposure

We attempted to determine whether chrysotile lung burden in mesothelioma cases is related to cumulative exposure as determined by Equations 4, 5.

The regression has limited power, but it still provides statistically significant estimation:


Chrysotile=0.03CE−5.36, (10)

where Chrysotile—lung concentration of chrysotile fibers longer than 5 μm (mf/g dry lungs), and CE—cumulative chrysotile exposure by Equation 4 (f/cc-years) (*R* = 0.6, *R*^2^ = 0.36, *p* < 0.02).

Similar regression can be found for cumulative exposure estimated by Equation 5:


Chrysotile=0.017CE−5.22, (11)

where Chrysotile—lung concentration of chrysotile fibers longer than 5 μm (mf/g dry lungs), and CE—cumulative chrysotile exposure by Equation 5 (f/cc-years) (*R* = 0.6, *R*^2^ = 0.36, *p* < 0.02).

Based on Equations 10, 11, there is also a possibility of threshold, suggesting that chrysotile lung burden is not expected to be elevated if CE does not exceed 178–307 f/cc-years. This is an empirical value, with a non-statistically defined confidence interval. However, the possible central tendency threshold value is also indicative of the fact that threshold may exist for chrysotile and mesothelioma, and it can be driven by lung burden accumulation of fibers.

A similar regression equation can be constructed for tremolite asbestos concentrations in lungs:


Tremolite asbestos=0.466CE−241, (12)

where Tremolite asbestos—lung concentration of tremolite asbestos fibers longer than 5 μm (mf/g dry lungs), and CE—cumulative exposure by Equation 4 (f/cc-years) (*R* = 0.6, *R*^2^ = 0.36, *p* < 0.02).

Equation 5 would yield a similar relationship.

Based on Equation 12, tremolite asbestos concentration in lungs would not be expected to be elevated for total cumulative chrysotile fiber exposure of about 500 f/cc-years. Assuming that the tremolite asbestos fraction in exposure for Québec workers is on the order of 1% (27), it can be roughly estimated that a threshold value for tremolite asbestos is about 5 f/cc-years.

## 4 Discussion

The potency of chrysotile for the production of mesothelioma is a long-contested issue. Meta-analyses performed over the last few decades have suggested that chrysotile mesothelioma potency is significantly lower than that for commercial crocidolite and amosite, as well as for Libby asbestiform amphiboles (LAA) (7, 12, 14). Berman and Crump (28) demonstrated that the mesothelioma potency of chrysotile might not be statistically different from zero. The “tremolite asbestos hypothesis” that suggested that much of the mesothelioma potency of chrysotile can be attributed to amphibole asbestos contamination was never disproved (29). Korchevskiy et al. (30), nevertheless, demonstrated that non-zero, though very low, chrysotile potency can be derived from chemical composition and dimensions of fibers.

At the same time, some authors have questioned the quality of available exposure data for asbestos-related disease mortality in chrysotile-exposed cohorts (5). PCM-estimated exposure levels in the Québec chrysotile cohort were questioned as to possible bias caused by conversions from the available midget impinger “dust” data expressed in MPCF to membrane-filter fiber/cc counts.

Lenters et al. (5), in a study limited to lung cancer risk, proposed five “quality criteria” for chrysotile asbestos cohorts, suggesting that analysis restricted to studies with “few quality limitations of the exposure assessment component, the epidemiological evidence base is too sparse to draw conclusions about potency differences per fiber type.” Berman and Case (31), however, argued that there is no evidence that applying the Lenters et al. “quality criteria” to exposure values in asbestos epidemiology would change the estimated risk of lung cancer. Hodgson (32) also noted that limiting meta-analysis to the five “quality criteria” chosen by Lenters et al. (5) potentially erase important epidemiological evidence.

Of greatest importance for lung cancer, meta-analyses of chrysotile asbestos potency are most affected by two studies, one of which is the Québec cohort, the other, a study of South Carolina asbestos textile workers. The South Carolina cohort was assumed in some publications not to have had other amphibole asbestos exposures, despite evidence to the contrary from multiple lung-retained fiber studies. Nevertheless, omission of *either* of the two cohorts leads to “overreliance on a single study” (31, 33).

For mesothelioma, our study helps to ascertain both disease and exposure quality measurement factors and significance in the Québec study, which remains the chrysotile cohort with the largest number of mesothelioma cases recorded. Following the same logic as for lung cancer, we believe that exclusion of this cohort from meta-analysis (as was recently attempted by the U. S. EPA in TSCA risk evaluation for chrysotile) (6), would lead to a loss of significant epidemiological information and bias the outcome of risk analysis beyond any practical applicability.

The Québec cohort is also of interest when the question of threshold for exposure is considered. The existence of a threshold for asbestos exposure in mesothelioma is supported by various studies and approaches (as reviewed by Goodman et al., 2025) (9). The level of threshold exposure in the Québec study can be assessed from various positions, as well. In particular, the minimum level of exposure in mesothelioma cases (or lowest observed adverse effect level) can be useful for the overall argument, though not being a “proof” for threshold, but providing scientists and regulators with reasonable practical benchmarks of mesothelioma risk.

Our paper provides additional arguments for inclusion of data on mesotheliomas in the Québec miners and millers cohort in meta-analysis for chrysotile potency. We utilized the originally published datapoint pairs (22) for parallel analysis of midget impinger and PCM measured concentration for the Québec asbestos industry that were not previously available for this type of analysis. Regression equations were determined between fiber concentrations as dependent variable and midget impinger concentration as predictor. If all original datapoints from Dagbert were utilized as published, the regression is statistically significant, but with comparatively low correlation coefficient (*R* = 0.46, *R*^2^ = 0.21, *p* < 0.00001). However, if concentrations by midget impinger and PCM values are averaged by groups of 30 datapoints, the correlation improves (*R* = 0.91, *R*^2^ = 0.82, *p* < 0.000001). We assumed that both regressions in our study can be used in comparisons, providing estimations for fiber concentration based on MPCF values.

We suggested that the application of log–log regression equation based on the original data provides a cumulative exposure estimation for the cohort that is not substantially different from what was used previously, and likely even higher than previous estimations. The average cumulative exposure of 528.5 f/cc-years (95% CI 510.3, 546.7) based on Equation 4 in our study and 979 f/cc-years (95% CI 945, 1,013) based on Equation 5 are of the same range as previously published estimates of 600 f/cc-years.

We performed a statistical simulation and demonstrated that both Equations 4, 5 can serve for estimation of average potency for selection of datapoints, similar to the dataset we used on our study. In particular, all average values of chrysotile estimated by Equation 4 are expected to be lower than observed by a factor of 2 and greater. The corresponding ratio for Equation 5 will be closer to 1, with 0.8 being the lower bound. This means that risk estimations based on Equation 4 will be always conservative, with at least a safety factor of 2; however, Equation 5 provides a better estimation of mesothelioma risk factors.

Our work allows us to update the estimation of chrysotile potency that Darnton published in 2023–2024 (7, 14). In addition, we included a cohort from Russia published by IARC to meta-analysis (this cohort was not considered in Darnton’s work). Utilization of Equation 5 (that was demonstrated to better approximate exposure values) put the estimated chrysotile potency at the level of 0.0010–0.0011%, vs. 0.0014% in Darnton (7, 14). If Equation 4 were used, the overall chrysotile potency would be about 0.0017%, but this would include the potency of Québec chrysotile over-estimated by a factor of 2 and higher.

We also explored implications of our approach for determination of a “practical threshold” related to chrysotile exposure and mesothelioma. The LOAEL estimation of minimal cumulative exposure in mesothelioma cases demonstrated values that ranged from 4.8–6 MPCF-years, or 18–50 f/cc-years.

We also performed a statistical simulation to generate larger intervals of cumulative exposure values in correspondence with mesothelioma rate. The linear-log model was utilized. Based on the developed models we described, the average threshold value is 6.38 MPCF-years (standard deviation 2.6), or in the range from 35.9 f/cc-years (standard deviation of 15.4) to 65.9 f/cc-years (standard deviation of 29.6).

A simulation with random combination of datapoints would suggest even higher threshold levels for average cumulative exposure. In particular, no mesothelioma cases in the Québec cohort were determined for average cumulative exposure levels of 113 MPCF-years by impinger, 309 f/cc-years by PCM Equation 4, or 577 f/cc-years by Equation 5.

Analysis of lung fiber burden, with values derived from McDonald et al. (4) and using the methodology therein, also confirmed that concentrations of fibers in lung tissue can be related to cumulative exposure, estimated based on the proposed log–log regression equation. Our study has demonstrated that the central tendency estimation when chrysotile lung burden concentration is used in this way as exposure proxy is not expected to be elevated for cumulative exposure below about 178–300 f/cc-years, nor tremolite asbestos below about 5 f/cc-years.

It should be noted that chrysotile does accumulate in the lungs of miners and millers, as does tremolite. Unlike the situation in experimental animals, where it has been shown that rapid clearance of chrysotile takes place after exposure, these workers were largely exposed to very high concentrations of chrysotile for decades, with resulting lung accumulation of chrysotile fibers (4).

Table 6 contains data on clearance rates for chrysotile asbestos in human lungs in comparison to other types of minerals, based on the summary from various sources as demonstrated in Korchevskiy and Wylie (34).

**Table 6:** Average published clearance rate of chrysotile and other mineral types of asbestos in human lungs.

Mineral type	The average published estimation (years^−1^) (Range)
Chrysotile	6.36 (0.086–25.3)
Crocidolite	0.092 (0.01–0.166)
Amosite	0.19 (0.03–0.46)
Tremolite	0.14 (0.08–0.19)

As we can see, chrysotile is expected to be quickly cleared from human lungs, and tremolite to stay there much longer. It is noteworthy that in the lungs of Québec workers, chrysotile was observed at substantial levels, that can be the indicator of slowed clearance rate, or continuous exposure up to the moment that the biopsy was performed. It is possible, however, that even if chrysotile fibers remained in the lungs of workers, the level of lung burden might be below a threshold level that would be sufficient to increase the risk of disease.

Interestingly, meta-analysis by Goodman et al. (9) suggested that, if threshold for chrysotile exists, it can be found at the level of 90 f/cc-years, and tremolite asbestos “threshold” in the range from 4.3 to 10.8 f/cc-years.

Additional studies would be needed to perform a full sensitivity analysis for the relationship between threshold level and various assumptions. However, consistency of threshold estimations between various recent publications is encouraging that at least “practical thresholds” for various mineral types of asbestos can be derived from available data.

As we can see, it was demonstrated in our study that more robust estimations of cumulative exposure for the Québec cohort are not expected to significantly change estimations of meta-analytical potency factor as calculated by Darnton, even if new IARC estimation of mesothelioma mortality in Russian chrysotile cohort would be included to the analysis. We demonstrated that mesothelioma potency for all types of chrysotile can be estimated as 0.0014% (95% CI 0.0010, 0.0.0018), and for non-textile chrysotile 0.0011% (95% CI 0.0008, 0.0015), fully confirming Darnton’s analysis.

Several approaches allowed to suggest different estimations of possible threshold on mesothelioma risk depending on cumulative exposure. Our analysis cannot demonstrate existence of a threshold but can suggest what the estimation should be if existence of threshold is proven on a biological basis. The range of estimation is quite wide. An especially important observation is that the detected threshold on average exposure in a cohort can be significantly higher than for individuals. The reason for this difference is related to the fact of uncertainties of possible alternative exposures (as well as variability in spontaneous mesothelioma rates) in the cohort members that is smoothed out by average cumulative exposure estimated for a group of people. Analyzing Table 1, we can see that among non-textile chrysotile cohorts, the Russian study shows the lowest average exposure producing elevated mesothelioma level in a cohort (33.6 f/cc-years). However, even much higher average exposure (120 f/cc-years) apparently has not provided elevated risk of mesothelioma (zero excess mesothelioma cases in Qinghai). In this context, we can see that true threshold for chrysotile cumulative exposure in mesothelioma can be quite substantial (if its existence would be fully demonstrated, as it is possible to hypothesize based on biological mechanisms of carcinogenicity).

Our study has uncertainties and limitations. We have mentioned “tremolite,” which is an amphibole that occurs in varying concentration in the mines in Québec in both asbestiform and nonasbestiform habits. There has been much written as to whether the tremolite—rather than the chrysotile—is responsible to a greater or lesser extent for mesothelioma and other asbestos-related diseases in the cohort, and indeed McDonald et al. (4) have shown this to be true for cohort members with more than 20 years’ service in those areas with higher tremolite content. However, as recently pointed out by Chatfield (27), while there is considerable variation between different mines, “It appears that “tremolite-free Canadian chrysotile” does not exist,” and that all Canadian sources analyzed contain tremolite/actinolite, at least “a proportion of which is asbestiform in morphology.” Therefore, it is not possible to assign all the risk for mesothelioma only to tremolite; the possibility of chrysotile itself providing risk—at very high dose—remains.

Our study demonstrated that a relationship between cumulative exposure to chrysotile and lung fiber burden can be established, and that the corresponding regression equation may have a threshold, with a certain accumulated number of fibers needed to increase the probability of disease. Relationship between cumulative exposure and lung fiber burden has been well established in scientific literature (34–37). However, for chrysotile workers in Québec, only limited data exists to evaluate this type of relationship. It seems that a lung burden study for the Québec population could be beneficial to confirm the conclusion of our modeling attempt.

A major question addressed in this study was our effort to improve the degree to which historic dust measurements taken by midget impingers, in MPCF, can be reliably used as proxy for fibers within the dust. Overall, it remains the case that the proportion of fibres among total dust “particles” is unknown. However, the new analyses and methods presented here using Dagbert’s paired sample data (23) from a variety of mines, mills, and jobs within them have improved our understanding.

Mesothelioma diagnosis has advanced considerably since the days during which every one of our cases occurred. Immunohistochemical tests specifically identifying mesothelial cell origin only became available in the late 1990s (10). We have therefore taken a conservative approach in our analysis and excluded three cases (of 30 overall) with low diagnostic probability. The possibility of inclusion of cases which were not mesothelioma is also balanced by the fact that mesothelioma has historically been under-diagnosed due to lack of awareness by pathologists and clinicians as well as anomalies in death certification.

Additional efforts are needed to explore the correspondence between Québec cohort study datapoints and the newly acquired specific measurements published in a governmental report by Dagbert in 1976 (23) and used here. However, our simulation was intended to cover various random combinations of exposure locations that can be attributed to the parallel measurements performed by Gibbs (38). The empirical relationship between mesothelioma mortality and cumulative exposure appears to depend on the selection of exposure categories (ranges). Further work is needed to test this relationship for different ranges of exposure. It may be beneficial if the Québec cohort can be followed for an additional time period, since the youngest surviving members were aged 73 at last follow-up. Nevertheless, our study addressed various aspects of the reliability of historical exposure data for chrysotile miners and millers and confirmed that possible statistical variability of exposure estimates should not significantly affect slope factors used for scientifically based risk assessment. Asbestos science continues to bring important methodological outcomes that would be valuable for epidemiology, toxicology, and other areas of research.

## Data Availability

The original contributions presented in the study are included in the article/supplementary material, further inquiries can be directed to the corresponding author/s.

## Glossary

Asbestiform habit: A morphology of minerals that have been naturally crystallized to be easily separated into long, thin, flexible, and durable fibers (39).
Non asbestiform habit: Mineralogical growth forms that produce elongate mineral particles (EMPs) when fragmented by mechanical force (39).
Tremolite asbestos: Non-commercial type of asbestos, a member of the amphibole group with a composition Ca_2_Mg_5_(Si_8_O_22_)(OH)_2_.
Chrysotile: Mineral form of commercial asbestos, a member of serpentine group, with a chemical formula Mg_3_(Si_2_O_5_)(OH)_4_.
PCM (phase-contrast microscopy): A method used for counting fibers by using a filter, analyzed by a phase-contrast microscope.
Midget impinger: A device used to count particles collected in a liquid medium.
MPCF: Million particles per cubic foot; unit of particle count by midget impinger.
