# Correction: Updates on exposure estimation for the Québec cohort of chrysotile miners and millers: implications for risk assessment and threshold hypothesis

**DOI:** 10.3389/fpubh.2026.1787841

**Published:** 2026-02-23

**Authors:** Bruce W. Case, Andrey A. Korchevskiy, Arseniy Korchevskiy

**Affiliations:** 1 Department of Pathology, McGill University, Montreal, QC, Canada; 2 Chemistry and Industrial Hygiene, Inc., Lakewood, CO, United States

**Keywords:** mesothelioma, chrysotile, Québec cohort, midget impinger, PCM

In the **Acknowledgments**, there was a mistake. The published version was: “The authors acknowledge Debbie Vaughan (Chemistry & Industrial Hygiene, Inc.) for technical support in organization and processing the database information, and preparation of the paper for publication. The authors are grateful to Igor Burstyn for valuable collaboration and discussion on the materials that were used in this paper and on the methodology of the analysis, along with submitting and providing the IRB assessment for the project “Epidemiological evidence of a threshold in associations of lung cancer in the Québec cohort of chrysotile miners and millers” that was used in conjunction with the studies described in this paper.” The acknowledgment should read as follows: “The authors acknowledge Debbie Vaughan (Chemistry & Industrial Hygiene, Inc.) for technical support in organization and processing the database information, and preparation of the paper for publication.”

A correction has been made to the section “**Materials and methods**,” last paragraph. The statement “The study was determined to be not human subject research by the Institution Review Board of Drexler University” has been removed.

The **Ethics statement** was originally given as “Ethical approval was not required for the study involving humans in accordance with the local legislation and institutional requirements. Written informed consent to participate in this study was not required from the participants or the participants' legal guardians/next of kin in accordance with the national legislation and the institutional requirements.” The amended **Ethics statement** is “Ethical approval was not required for the study involving humans in accordance with the local legislation and institutional requirements. The study was found to qualify for exemption from IRB review under the federal regulations outlined in 45 CFR 46.104 (d), according to the assessment by the Beyond Bound IRB (IRB ID # BB2512JD-170). Written informed consent to participate in this study was not required from the participants or the participants' legal guardians/next of kin in accordance with the national legislation and the institutional requirements.”

The original version of this article has been updated.

